# OXA-48 Carbapenemase in *Klebsiella pneumoniae* Sequence Type 307 in Ecuador

**DOI:** 10.3390/microorganisms8030435

**Published:** 2020-03-19

**Authors:** José E. Villacís, Jorge A. Reyes, Hugo G. Castelán-Sánchez, Sonia Dávila-Ramos, Miguel Angel Lazo, Ahmad Wali, Luis A. Bodero, Yadira Toapanta, Cristina Naranjo, Lorena Montero, Josefina Campos, Marcelo G. Galas, Mónica C. Gestal

**Affiliations:** 1 Facultad de Medicina, Pontificia Universidad Católica del Ecuador, Quito 170143, Ecuador; 2 Centro de Referencia Nacional de Resistencia a los Antimicrobianos, Instituto Nacional de Investigación en Salud Pública “Leopoldo Izquieta Pérez”, Quito 170403, Ecuador; 3 Facultad de Ciencias Químicas, Universidad Central del Ecuador, Quito 170129, Ecuador; jorgereyes83@gmail.com; 4 Centro de Investigación en Dinámica Celular, Instituto de Investigaciones Básicas y Aplicadas Universidad Autónoma del Estado de Morelos, Cuernavaca Morelos 62209, Mexico; hcastelans@gmail.com (H.G.C.-S.); sonia.davila@uaem.mx (S.D.-R.); 5 Hospital de Especialidades Eugenio Espejo, Quito 170136, Ecuador; miguellazo2001@gmail.com (M.A.L.); oncowali@yahoo.es (A.W.); yadybioclin@hotmail.com (Y.T.); mb.cristina.naranjo@hotmail.com (C.N.); 6 Ministerio de Salud Pública, Quito 170702, Ecuador; luisandresbd@gmail.com (L.A.B.); lpmonterot@gmail.com (L.M.); 7 Universidad San Francisco de Quito, COCIBA, Instituto de Microbiología, Quito 170901, Ecuador; 8 Administración Nacional de Laboratorios e Institutos de Salud ”Dr. Carlos G. Malbrán,” Buenos Aires 1281, Argentina; jocampos05@gmail.com; 9 Pan American Health Organization/WHO, Washington, DC 20037, USA; galasmar@paho.org; 10 Louisiana State University (LSU), Health Science Center, Shreveport, LA 71103, USA

**Keywords:** *Klebsiella pneumoniae*, OXA-48 carbapenemase, carbapenemase-producing Enterobacterales

## Abstract

Antibiotic resistance is on the rise, leading to an increase in morbidity and mortality due to infectious diseases. *Klebsiella pneumoniae* is a Gram-negative bacterium that causes bronchopneumonia, abscesses, urinary tract infection, osteomyelitis, and a wide variety of infections. The ubiquity of this microorganism confounds with the great increase in antibiotic resistance and have bred great concern worldwide. *K. pneumoniae* sequence type (ST) 307 is a widespread emerging clone associated with hospital-acquired infections, although sporadic community infections have also been reported. The aim of our study is to describe the first case of *Klebsiella pneumoniae* (ST) 307 harboring the blaOXA-48-like gene in Ecuador. We characterized a new plasmid that carry OXA-48 and could be the source of future outbreaks. The strain was recovered from a patient with cancer previously admitted in a Ukrainian hospital, suggesting that this mechanism of resistance could be imported. These findings highlight the importance of programs based on active molecular surveillance for the intercontinental spread of multidrug-resistant microorganisms with emergent carbapenemases.

## 1. Introduction

Antibiotic resistance is considered a XXI century pandemic [1], and several federal and governmental organisms, such as the World Health Organization (WHO), are calling for an urgent action to halt the spread the multidrug resistance microorganisms [2]. Carbapenem-resistant Enterobacterales are included in the list of urgent threats provided by the CDC (https://www.cdc.gov/drugresistance/biggest-threats.html [3]), and its dissemination throughout the world is a major public health worry. Carbapenemases are enzymes capable of hydrolyzing almost all β-lactam antibiotics and they are commonly located in plasmids, allowing to be spread throughout a great diversity of Gram-negative bacteria [4,5]. Serine carbapenemases (KPC) and metallo-β-lactamases (NDM, IMP, VIM) are the most prevalent carbapenemases reported all around the globe [6], KPC being the first to be described in 2001 in an isolate of *Klebsiella pneumoniae* in USA [7]. Carbapenem-resistant isolates within *K. pneumoniae* have been spread globally [8,9,10,11], including clone 307, which has significantly increased in the mid-1990s and is associated with several resistance genes, such as OXA-48 [12,13].

OXA-48-like carbapenemase was first described in Turkey in 2004 in a *K. pneumoniae* strain [8], and since then it has been reported in Europe, North Africa, and the USA [14,15,16,17]. Nowadays, several outbreaks have been registered in many South America countries, including Colombia [18], Argentina [19], and Brazil [20]. There are 11 allelic variants of OXA-48-like that have been described up to now [21]. The gene *bla*OXA-48 is located in 60- to 70-kb conjugative plasmids, generally associated with the transposon Tn*1999* and its variants *Tn1999.2* and *Tn1999.3*, and in the majority of the published data it has been associated with a nearby copy of *IS1R* [18,22,23]. Interestingly, OXA-48-like β-lactamases hydrolyzes penicillins and carbapenems but spares extended-spectrum cephalosporins. Here we report the first case of *Klebsiella pneumoniae* ST 307 carrying the *bla*OXA-48-like carbapenemase gene in Ecuador, and we use whole-genome sequencing (WGS) to characterize the carrying plasmid.

## 2. Materials and Methods

### 2.1. Patient History

In August 2016, a woman with colorectal cancer status—post two operations—in Ukraine was admitted to the Eugenio Espejo Hospital in Quito, Ecuador, for palliative treatment. Twenty days later, an ascitic fluid sample was processed at the microbiology laboratory of the hospital. An isolate of *Klebsiella pneumoniae* was identified using the VITEK^®^2 GN ID card (bioMerieux Inc., France).

### 2.2. Antimicrobial Susceptibility

Drug susceptibility was tested using the Vitek-2 compact system (bioMerieux Inc., France) utilizing the AST N272 card. Furthermore, the isolated sensititre (Trek Diagnostic Systems, UK) GNX2F microdilution broth plates and Modified Carbapenem Inactivation Methods (mCIM) and EDTA-modified carbapenem inactivation method (eCIM) were performed to differentiate metallo-β-lactamases from serine carbapenemases [24]. The results were interpreted according to the Clinical and Laboratory Standards Institute [24].

The isolate was sent to the National Reference Laboratory of Antimicrobial Resistance (NRLAR) “Dr. Leopoldo Izquieta Pérez”, Quito, for further investigation. At the NRLAR, we utilized molecular approaches to determine the mechanism of resistance.

### 2.3. DNA Extraction and Polymerase Chain Reaction

DNA was extracted following the recommendations of the manufacturer DNA Wizard (Promega). Polymerase chain reaction (PCR) was used to identify carbapenem resistance genes (blaKPC, blaIMP, blaNDM, blaVIM, and blaOXA-48-like) [25]. Multilocus sequence typing (MLST) analysis was performed using seven housekeeping genes (*gapA*, *infB*, *mdh*, *pgi*, *phoE*, *rpoB*, and *tonB*) [26], as recommended by the Pasteur Institute scheme (http://www.pasteur.fr).

### 2.4. Conjugation Assays

To demonstrate the horizontal gene transference, conjugation assays were performed using the broth-mating technique with an azide-resistant *Escherichia coli* J53 as a recipient [27,28,29]. The plasmid incompatibility group was determined using a PCR-based replicon typing (PBRT) kit (Diatheva, Fano, Italy) [30]. The *IS*1999 element was amplified and linked with the *bla*OXA-48-like gene using the primers reported previously [31].

### 2.5. Whole Genome Sequencing and Bioinformatic Analysis

Whole Genome Sequencing (WGS) was executed at the Institute Malbran using Illumina Miseq (https://www.illumina.com) for paired-end reads; the quality of reads was evaluated using a FastQC version [32], and adapters were eliminated with PEAT [33]. The reads below a quality 25 Phred score were eliminated with Trim Galore [34]. The assembly was performed with Spades [35], and the quality of the assembly was evaluated with Quast [36]; our statistics are based on contigs of size ≥ 500 bp. The identity of the strain was verified using the standard 16s ribosomal, *groEL*, *rpoB*, and *recA* genes in the Genome Peek online tool [37]. The functional annotation was made with Prokka [38] and the NCBI Prokaryotic Genomes Automatic Annotation Pipeline [39] (http://www.ncbi.nlm.nih.gov/genome/annotation_prok) and eggnog mapper [40].

From the assembly, we search for the presence of plasmids using Plasmidfinder [41]. To identify the OXA-48-like plasmid sequence, we mapped our sequence against the reference sequence CP034283.1, both sequenced with PacBio RSII and Illumina NextSeq. Coverage was evaluated using BBmap [42], and the consensus sequence was extracted using UGENE [29]. The plasmid map was obtained with BLAST Genome Atlas [43].

## 3. Results and Discussion

### 3.1. OXA-48 was Identified a K. pneumoniae MLST ST307 Isolate

The Ecuadorian surveillance network aims to provide solid data regarding epidemiological observation of antibacterial resistance. A *Klebsiella pneumoniae* strain with a high-resistance profile to imipenem, meropenem (intermediate), ertapenem (resistance), and piperacillin/tazobactam (intermediate), and susceptible to the third generation of cephalosporins (Table 1) was sent to the reference laboratory in order to determine the molecular mechanism of resistance associated with this phenotype. When studying inhibition to determine possible mechanisms of resistance, the mCIM resulted positive and eCIM was negative [24], suggesting that a carbapenemase Class D might be responsible for the identified phenotype. To further explore this hypothesis, Polymerase Chain Reaction (PCR) of several carbapenemases encoding genes revealed the presence of the *bla*OXA-48-like gene (Genebank accession number: KY609322.1).

Next, we wanted to investigate if this strain belongs to a previously described epidemic clone. Multilocus sequence typing (MLST) analysis using the Pasteur Institute scheme (http://www.pasteur.fr) as reference, revealed that the isolate belongs to the Sequence Type (ST) 307 (*gapA*: 4; *infB*: 1; *mdh*: 2; *pgi*:52; *phoE*:1; *tomb*: 7; *rpoB*:1). Interestingly, this clone was not previously reported in Ecuador, suggesting that this could be the first of many isolates. Importantly, one of the main characteristics of OXA-48 is its mobility, and this particular clone has been correlated with the presence of mobile elements.

### 3.2. pOXA-48 is Found on the HEEJev_01 Plasmid

To investigate if the mechanism of resistance was located in the plasmid or in the chromosome, conjugation assays were performed [44], revealing that the *bla*OXA-48-like gene was in the transconjugant strains suggesting that a plasmid was carrying the *bla*OXA-48-like resistance gene. The results were further corroborated using phenotypic (antimicrobial susceptibility) and molecular (PCR) tests (Table 1).

To determine if the plasmid also contained mobile elements that would allow to more strikingly disseminate, PCR assays were performed. PCR-based replicon typing (PBRT) from the recipient *E. coli* J53 (*12*) showed the presence of IncF, IncH, and IncL plasmid groups.

**Figure 1:**
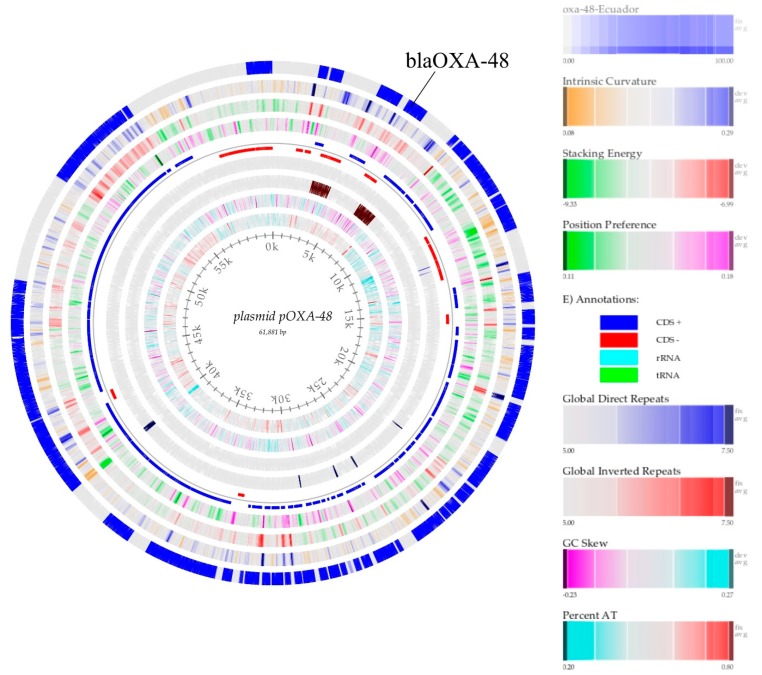
From center to outside, the circular map of plasmid pOXA-48 reference CP034283.1 outside in blue plasmid, pOXA-48 from Ecuador, intrinsic curvature, stacking energy, position preference, CDS+, CDS-, global inverted repeats, GC content, GC skew, and percent AT.

**Figure 2:**
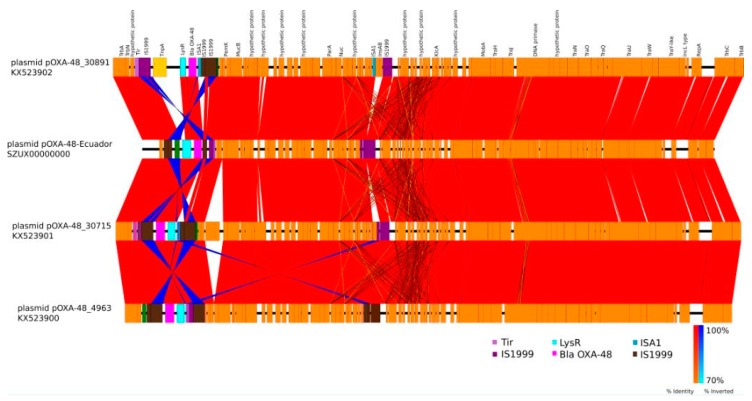
Plasmid OXA-48 of *K. pneumoniae* of Ecuador. Whole sequence shows a high degree of identity with plasmids from Czech Republic.

Although sequencing results were somehow limited, we were able to identify the presence of three independent plasmids, namely IncFIB (Mar), IncHI1B, and IncL/M(pOXA-48), further verifying the results obtained with PCR-based replicon typing (Table 1). Since it is the first isolate of *Klebsiella pneumoniae* ST 307 containing the pOXA-48 plasmid isolated in our country (Genebank accession number: SZUX00000000), we aimed to reconstruct the pOXA-48 plasmid using fragment recruitment, and the reference sequence CP034283.1 (Figure 1) plasmid that was obtained completely by sequencing in PacBio. Subsequently, our plasmid was compared with the reference plasmids KX523902, KX523901, and KX523900. We chose to compare it with these plasmids because according to a phylogenetic analysis (data not shown), the OXA-Ecuador plasmid was grouped together with these sequences. Thus, we were able to identify the sequence of the *bla*OXA-48-like gene and the IS transposons. Interestingly, the IS transposons were inverted when comparing the strains (Figure 2), suggesting that this plasmid is still evolving, and that the genes are rearranging to maybe increase its transmission and fitness.

## 4. Discussion

With the rapid increase of sophisticated mechanisms of resistance, the need for developing new methods to allow their identification is imperative [45,46]. Although Enterobacterales are sensitive to third and fourth generation cephalosporins and with intermediate sensitivity to imipenem/meropenem and resistance to ertapenem are uncommon and difficult to identify [47], the mCIM and eCIM appear to be suitable methods for carbapenemase screening in healthcare centers where molecular techniques are lacking [48,49]. There is evidence that *K. pneumoniae* ST307 has been described as a high-risk clone [50,51,52].

The ST307 genome contains some virulence genes, suggesting that this clone might harbor the necessary tools to confer advantages in the hospital environment [50]. For example, glycogen synthesis can provide long-term survival and growth in environments outside the host as it increases biofilm formation, which has been correlated with nosocomial pathogens [13,53,54,55]. Moreover, the presence of the IncF plasmid, which is spread among the Enterobacterales, was identified, suggesting that this may be involved in the diffusion of resistance determinants and, with a combination of certain high-risk clones, contribute to the dissemination of *AMR* genes [5,56], which has been previously reported for *K. pneumoniae* [57,58]. From a search of the Institute Pasteur database (https://bigsdb.pasteur.fr/klebsiella/; 3987 STs last updated 10 April 2019), 35 human and 1 environmental isolate around the world were ST307 and 12 isolates from Europe and 1 from Asia were found in human samples with carbapenemase blaOXA-48-like [50]. We recently reported a strain of *Raoutella ornithinolytica* that harbored the blaOXA-48-like gene with no plasmid characterization that was recovered after this strain [59], and to the best of our knowledge, this is the first description of carrying OXA-48-like carbapenemase in an IncL plasmid isolated in Ecuador. Interestingly, our patient came from Croatia were this mechanism of resistance was reported previously [60,61,62], indicating that contagion with this strain most likely happened in Europe. The implications of globalization and free travel are now being revealed with the fast spread of the SARS-CoV-19, but in reality, this is nothing new and it has long been the case with mechanisms of resistance. Overall, these results highlight the implications of globalization, supporting previously published data from here and other countries [59,63,64,65,66]. Altogether this suggests that global guidelines for antibiotic stewardship might need to be implemented in order to better control the dissemination of the current increasing antibiotic resistance. This type of emergent resistance mechanism has an important risk as it can be easily spread between different environments, especially hospital settings. Further research will be performed in order to monitor this strain and others that can carry this and other mechanisms of resistance.

## 5. Conclusions

Early detection and establishment of immediate control measures are key to control dissemination. From a public health perspective, the existence of an active national surveillance system for multidrug-resistant organisms would allow for early warnings and a subsequent quick ascertaining of the magnitude of the problem at the national level. Programs based on molecular surveillance, infection control, and antibiotic stewardship are required in hospitals of this country and all other countries, to detect emerging resistance genes as well as high-risk clones, and halt them.

## Figures and Tables

**Table 1:** Minimal concentration inhibitory of the *Klebsiella pneumoniae* OXA-48 producer and *Escherichia coli* J53 transconjugant.

MIC (μg/mL) *	*K. pneumoniae* OXA-48-like	*E. coli J 53* Transconjugant
IMI	2 (Intermediate)	2 (Intermediate)
MER	2 (Intermediate)	<1 (Susceptible)
ERT	4 (Resistant)	4 (Resistant)
CAZ	<1 * (Susceptible)	<1 (Susceptible)
CTX	<1 * (Susceptible)	<1 (Susceptible)
FEP	<2 * (Susceptible)	<2 (Susceptible)
ATM	<2 (Susceptible)	<2 (Susceptible)
PIP/TAZ	>64/4 (Intermediate)	>64/4 (Intermediate)
CIP	>2 (Resistant)	<0.25 (Susceptible)
COL	0.5 (Susceptible)	0.25 (Susceptible)
AK	<4 (Susceptible)	<4 (Susceptible)
GEN	8 (Intermediate)	8 (Intermediate)
TGC	<0.25 (Susceptible)	<0.25 (Susceptible)
mCIM	POS	POS

* Susceptible to third and four generation of cephalosporins. IMI: imipenem; MER: meropenem; ERT: ertapenem; CAZ: Ceftazidime; CTX: Cefotaxime; FEP: cefepime; ATM: aztreonam; PIP/TAZ: piperacillin/tazobactam; CIP: ciprofloxacin; COL: colistin; AK: amikacin; GEN: gentamicin; TGC: Tigecyclin; mCIM: modified carbapenem inactivation method.
